# Sulfated Seaweed Polysaccharides as Multifunctional Materials in Drug Delivery Applications

**DOI:** 10.3390/md14030042

**Published:** 2016-02-25

**Authors:** Ludmylla Cunha, Ana Grenha

**Affiliations:** 1 ludmyllacc@gmail.com; 2

**Keywords:** carrageenan, drug delivery, fucoidan, macrophage targeting, ulvan, sulfated polysaccharides

## Abstract

In the last decades, the discovery of metabolites from marine resources showing biological activity has increased significantly. Among marine resources, seaweed is a valuable source of structurally diverse bioactive compounds. The cell walls of marine algae are rich in sulfated polysaccharides, including carrageenan in red algae, ulvan in green algae and fucoidan in brown algae. Sulfated polysaccharides have been increasingly studied over the years in the pharmaceutical field, given their potential usefulness in applications such as the design of drug delivery systems. The purpose of this review is to discuss potential applications of these polymers in drug delivery systems, with a focus on carrageenan, ulvan and fucoidan. General information regarding structure, extraction process and physicochemical properties is presented, along with a brief reference to reported biological activities. For each material, specific applications under the scope of drug delivery are described, addressing in privileged manner particulate carriers, as well as hydrogels and beads. A final section approaches the application of sulfated polysaccharides in targeted drug delivery, focusing with particular interest the capacity for macrophage targeting.

## 1. Introduction

Marine environment and the associated wide diversity of organisms offer a rich source of valuable materials. Among marine resources, algae, which are sometimes referred as seaweeds, are well known natural sources of polysaccharides. Sulfated polysaccharides are of the most common in the cell walls of seaweeds. The number and chemical structure of these polymers vary according to the specific algal species [1].

Marine algae can be classified into three main groups based on the exhibited photosynthetic pigments: red, brown and green. Botanists refer to these groups as Rhodophyceae, Phaeophyceae and Chlorophyceae, respectively. Brown seaweeds are usually large and range from the giant kelp that is often 20 m long, to thick, leather-like seaweeds of 2–4 m long, to smaller species 30–60 cm long. Red seaweeds are usually smaller, generally ranging from a few centimeters to about a meter in length. Curiously, red seaweeds are not always red, sometimes being purple, even brownish red, but still being classified by botanists as Rhodophyceae because of other characteristics. Green seaweeds are also small, with a size range similar to that of red seaweeds [2].

**Figure 1:**
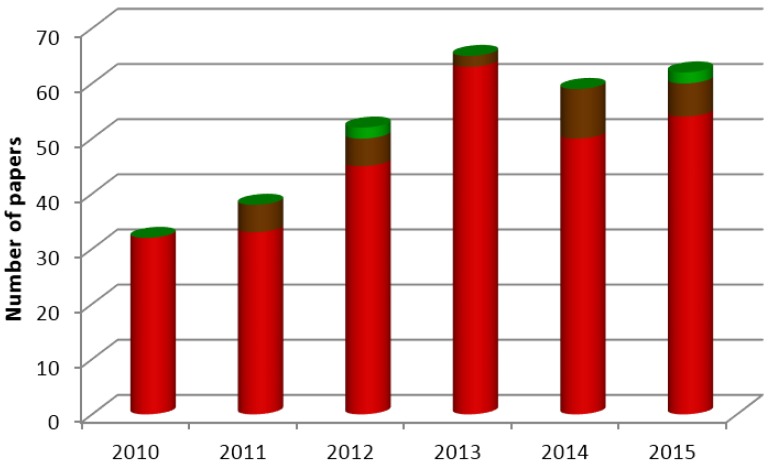
Number of scientific publications published on the topic “name of polymer” and “drug delivery” as a function of publication years. Taken from ISI Web of Knowledge. The colors allude to the colors of algae (**red**: carrageenan, **brown**: fucoidan, **green**: ulvan).

In the last decades, sulfated polysaccharides of algal origin have attracted much attention as functional additives in the pharmaceutical field, but also in food and cosmetic industries. The major sulfated polysaccharides found in marine algae include carrageenan from red algae, ulvan isolated from green algae and fucoidan from brown algae [3]. Carrageenan is the most used of the three, with wide application as emulsifier, stabilizer or thickener. Fucoidan, in turn, is available commercially from various cheap sources and has been investigated in recent years to develop novel drugs, medicines and functional foods. Ulvan is the less known of the group. It displays several physicochemical and biological features of potential interest for food, pharmaceutical, agricultural and chemical applications, but needs deeper investigation. Various studies have revealed that sulfated polysaccharides isolated from marine algae exhibit a variety of biological activities [4,5,6], potentiating their use in pharmaceutical applications. These polymers have been increasingly studied over the years in this context, given their potential usefulness in applications that mainly involve the design of drug delivery systems. Figure 1 depicts the number of publications retrieved on ISI Web of Knowledge with the keywords “name of the polymer” and “drug delivery”, showing the increasing interest in carrageenan and fucoidan in the recent years, with the undoubted prevalence of the former. Additionally, it confirms that ulvan is the less explored of the three polymers.

The purpose of this review is to discuss potential applications of sulfated polysaccharides, with a special emphasis on carrageenan, ulvan and fucoidan, in designing drug delivery systems and also address their suitability for specific applications, such as cell targeting. A summary of basic characteristics of the three carbohydrates is displayed in Table 1.

## 2. Carrageenan: Sulfated Polysaccharide of Red Seaweeds

Historically, red seaweeds (Rhodophyta) have been harvested and consumed as foods for at least 2800 years. Although red algae are consumed by humans, the carrageenan extracted from seaweed is not assimilated by the human body, merely providing bulk. However, these algae do provide functional properties that are exploited on a commercial scale [23]. As one of its main properties relies on the ability to form thermoreversible gels or highly viscous solutions, carrageenan is commonly used as gelifying, stabilizing and emulsifying agent in food, pharmaceutical and cosmetic industry [24]. A broad and recent review on the industrial applications of carrageenan, including in food and pharmaceutical related areas, is available on [25].

### 2.1. Origin, Extraction and Processing

The original source of carrageenan was the red seaweed *Chondrus crispus* (also known as Irish Moss). With the expansion of the carrageenan industry over time, the increasing demand for the raw material led to the introduction of the cultivation of species of *Eucheuma*, originally *E. cottonii* and *E. spinosum*, now referred to as *Kappaphycus alvarezii* and *Eucheuma denticulatum*, respectively [2,23]. The advantage thereof compared to the natural *Chondrus crispus*, is a predominant content of *kappa*- and *iota*-carrageenan, respectively, while *Chondrus crispus* contains a mixture of *kappa* and *lambda* carrageenan that cannot be separated during commercial extraction. Therefore, most of carrageenan is now extracted from *K. alvarezii* and *E. denticulatum* but several species of *Gigartina*, *Iridae*, *Hypnea* and *Furcellaria* genera have been exploited, providing different types of carrageenan extracts [2,26,27].

Carrageenan manufacture consists of extraction, purification, concentration, precipitation and drying, although the basic process may vary according to the red algae family from which the polysaccharide is extracted. In some cases, pretreatment to remove excess of color or alkaline modification can be also performed prior to extraction [23]. The specific details of the extraction process are secured as trade secrets by several carrageenan manufacturers. There are two main methods for producing carrageenan based on different principles. Briefly, in the first and original method carrageenan is extracted from the seaweed in an aqueous solution. After filtration to remove the remaining residues, carrageenan is usually recovered from the solution by addition of an alcohol to induce precipitation. Finally, the precipitate is separated, dried and milled, resulting in a refined carrageenan. In the second method, carrageenan is actually not extracted from the seaweed. Instead, the principle is to wash out residual minerals, soluble protein and fat from the seaweed, leaving behind carrageenan and other insoluble matter. This insoluble residue, consisting largely of carrageenan and cellulose, is then dried and sold as semi-refined carrageenan, usually for non-food gelling applications. Although the process is much shorter and cheaper than the first one, its purity is necessarily lower [2,28,29,30,31]. Other methods can be found in the literature such as enzyme-treated or fungal-treated extractions [32,33,34,35]. Enzymatic extraction, for example, can enable the production of specific gelation properties, since the physicochemical properties of carrageenan depend on the composition of the polysaccharide and on the number of counterions [36].

### 2.2. Chemical Structure and Types of Carrageenan

**Figure 2:**
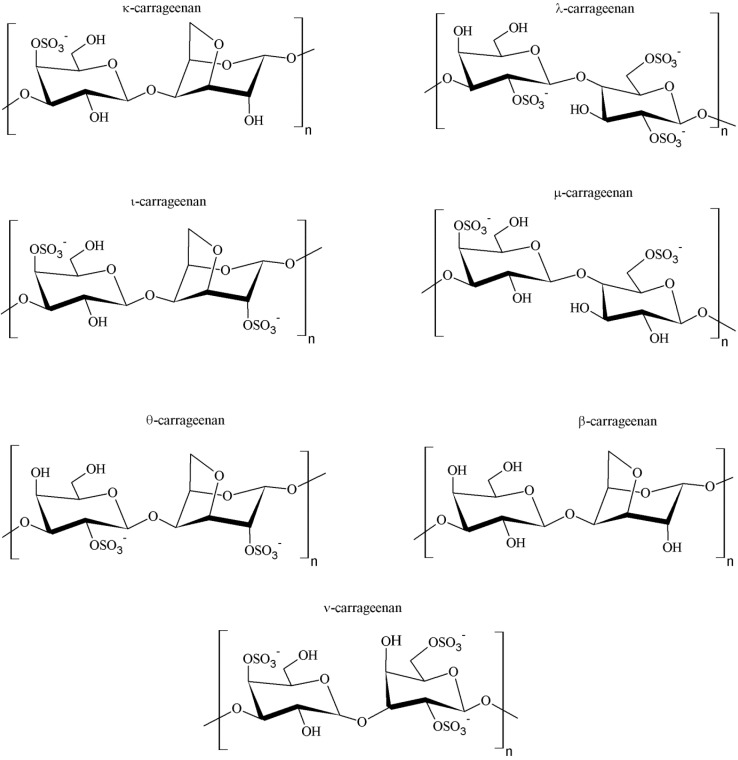
Carrageenan structures. Linear chains of repeating galactose units in d configuration and 3,6-anhydro-galactose copolymer, joined by alternating α-(1→3) and β-(1→4) glycosidic linkages.

The seaweeds that biosynthesize this polysaccharide are called carrageenophytes. Carrageenan is in fact a general name for a family of galactans, the commonest and most abundant cell wall constituents encountered in red algae. The backbone structure of this polysaccharide is based on linear chains of repeating galactose units in d configuration (d-sugar) and 3,6-anhydro-galactose copolymer, joined by alternating α-(1→3) and β-(1→4) linkages, as shown in Figure 2. In terms of chemical structure, this polygalactan is classified into various types, including but not limited to κ-, λ-, ι-, μ-, θ-, β- and ν-carrageenans, all containing 15%–40% ester sulfate with the exception of β-carrageenan, which is devoid of sulfate content [1,26].

At least 15 different carrageenan structures are reported, with *κappa* (κ), *ιota* (ι) and *lambda* (λ) forms being the most industrially relevant. The major difference among the various forms of carrageenan is related to structural characteristics, including the number and the position of sulfate groups and the occurrence of 3,6-anhydro-d-galactose in the chain [37]. For instance, κ-, ι- and λ-carrageenans are distinguished by the presence of one, two and three ester-sulfate groups per repeating disaccharide unit, respectively [38]. The chemical structures of carrageenans are, thus, very heterogeneous and are correlated to the algal sources, the life stage of the seaweed (*i.e.*, gametophyte) and the extraction procedures of the polysaccharide [27].

### 2.3. Physicochemical Properties

The particular composition and the conformation of a given polysaccharide constitute the basis of its physicochemical and biological properties. Self-assembling and gel formation ability, for instance, are related with specific conformations resulting from primary polysaccharide structures [38].

The average molecular weight of commercially available carrageenan ranges from 100 to 1000 kDa [9]. The structure of κ-carrageenan was reported as alternating 3-linked β-d-galactose and 4-linked anhydro-galactose (AG) units. It has an ester sulfate content of about 25%–30% and a 3,6-AG content of about 28%–35%. *Iota* carrageenan has an additional sulfate group on C-2 of the AG residue, resulting in two sulfates per disaccharide repeating unit. It has an ester sulfate content of 28%–30% and about 25%–30% content of 3,6-AG. *Lambda* carrageenan has three sulfate groups per disaccharide unit with the third sulfate group of this form at the C-6 position of the 4-linked residue. There is an ester sulfate content of about 32% to 39% and no 3,6-AG content [38].

Carrageenan exhibits the solubility characteristics normally shown by hydrophilic colloids. It is water soluble and insoluble in most organic solvents such as alcohol, ether and oil. Aqueous solubility is influenced by a number of factors, including the type of carrageenan, temperature, pH, number of counterions and the presence of other solutes. The numerous forms of carrageenan, showing variations in the chemical structure, as described above, provide much variability regarding solubility properties [8,23]. In general, hydrophilicity is structurally provided by the hydrophilic sulfate and hydroxyl groups, while 3,6-anhydro-d-galactose residues (3,6-AG) are more hydrophobic [39]. Therefore, λ-carrageenan, being highly sulfated and having no 3,6-AG content, is easily soluble under most conditions, whereas κ-carrageenan, with 3,6-AG residue and fewer sulfate groups, is relatively less hydrophilic and, thus, less soluble. The intermediate ι-carrageenan, is more hydrophilic due to the presence of the two sulfate groups, which counteract the slight hydrophobic character of the 3,6-AG residue. Solubility characteristics are also affected by the salt form of the sulfated ester groups. The free acid is unstable and, therefore, commercial carrageenans are available as sodium, potassium and calcium salts or, most commonly, as a mixture of these. These salts afford the needed stability. In general, the sodium forms of carrageenan are more easily soluble, while potassium forms dissolve with more difficulty. For instance, the potassium salt of both κ- and ι-carrageenan is insoluble in cold water, requiring the application of temperature to bring the polysaccharide into solution, whereas in the sodium form it dissolves readily. However, λ-carrageenan is soluble in all its salt forms. Moreover, both the dissolving rate and solubility of carrageenan are affected by the presence of other solutes which may compete for available water, thus altering the state of hydration of the polysaccharide [8].

The products emerging from the manufacturing process are inherently variable. Differences in algal sources and adjustments in processing conditions produce different carrageenans. The standardization of the product is determined by the functionality required in the specific application. The product is sold as a powder, which dissolves slowly at room temperature, producing a highly viscous aqueous solution. Viscosity depends on the type of carrageenan, its molecular weight, the used polymer concentration, applied temperature and the presence of other solutes (such as salts). The viscosity of a carrageenan solution decreases with decreasing concentration, sulfation or molecular weight and by increasing temperature. In addition, due to its swelling ability, carrageenan is difficult to disperse in water due to the formation of a film layer around each carrageenan particle. This leads to the formation of large agglomerates which make it very difficult for the water molecules to penetrate. Efficient dispersion can be achieved by high-speed mixing or by premixing the powder with inert matter, such as a sugar in a carrageenan/sugar mass ratio of 1:10. The presence of acid and oxidizing agents in solution may induce carrageenan hydrolysis leading to loss of physical properties through cleavage of glycosidic linkages. Such depolymerization is greatly accelerated by the presence of dissolved oxygen, high temperature and low pH. Therefore, in order to ensure minimum degradation during processing, high temperature short time processes are preferred. In this context, optimum stability of carrageenan occurs at pH 9, while severe degradation is known to occur at pH below 3.5 [8,23].

**Figure 3:**
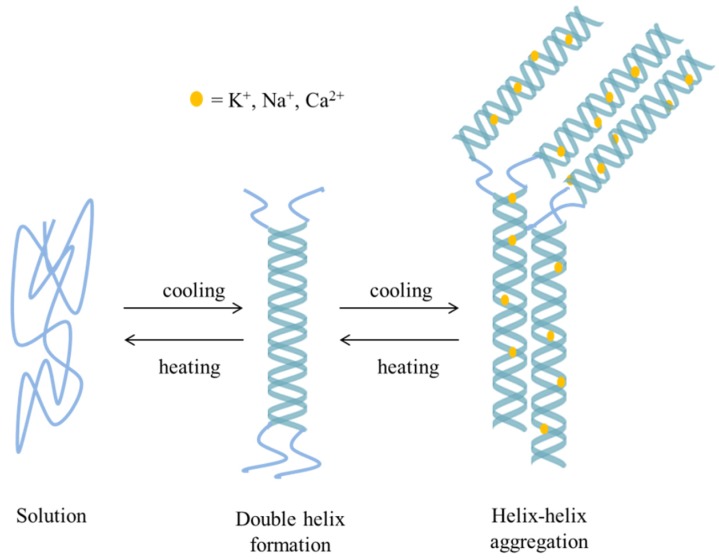
Scheme of carrageenan gel formation. The structure of κ- and ι-carrageenan allows segments of the two molecules to form the so-called double helices which bind the chain molecules in a three-dimension network. The associated counterions such as Na^+^, K^+^ and Ca^2+^, are also required to induce the sol-gel transition of the referred types of carrageenan.

As previously mentioned, commercial carrageenans are available as stable sodium, potassium and calcium salts. The associated cations together with the conformation of the galactose units in the polymer chain, which results in different carrageenan types, determine the gelling properties of these carbohydrates [8]. Thermal gelation is a valuable property of carrageenans that is determinant in diverse applications, including in food and pharmaceutical industries. The functionality of carrageenans in various applications depends on the rheological properties. Carrageenan solutions are non-Newtonian fluids and show pseudoplastic behavior [23]. At a certain range of temperatures and cation concentrations, carrageenan solutions may gel and the viscoelastic properties of these gels vary depending on these parameters [40]. The available carrageenans differ in their ability to undergo gelation. While κ- and ι-carrageenans form gels, λ-carrageenan does not gel and behaves as a common polyelectrolyte in solution [41]. There is general agreement on the mechanism of gelation of the polymer. It is assumed that, in solution and at high temperature, carrageenans exist as random coils. When dissolved by heating, followed by cooling below certain temperatures, the reduction in temperature induces the formation of double helices. The structure of κ- and ι-carrageenan allows segments of the two molecules to form the so-called double helices, which bind the chain molecules in a three-dimensional network that is in fact a gel. In turn, λ-carrageenan has a structure that does not allow the formation of such double helix, presumably due to its high degree of sulfate substitution, which hampers gel formation [40]. In other words, the gelation of carrageenan solutions occurs as a result of coil-to-helix conformational transition and the subsequent aggregation among ordered helices [42], as depicted in Figure 3. By itself, the formation of the helical structure does not lead to carrageenan gelation, though. The associated counterions such as Na^+^, K^+^ and Ca^2+^, mentioned before, are those responsible for the final sol-gel transition of the polysaccharide. The role of various cations in promoting cross-links and inducing a gelation was previously studied. In the particular case of carrageenan, the formation of the double helix corresponds to a limited number of chains that are linked together through intermolecular forces. However, this occurs into small domains that require further association by cation-mediated helix-helix aggregation to develop a cohesive network. Only the latter structure corresponds to the gel.

Of all carrageenans, *kappa* is the one providing the strongest gels. The strength of carrageenan gels is greatly dependent on the carrageenan concentration and is also related to the type and concentration of cation. The effectiveness of salts in influencing gel strength has been evaluated and it was shown that the addition of salts such as NaCl, KCl, CaCl_2_ and BaCl_2_ at an adequate concentration apparently improves the gel strength of κ-carrageenan gels by means of an enhancement of conformational ordering and subsequent aggregation. Several works devoted to the study of the influence of salts on carrageenan gel properties (κ- and ι-carrageenan) should be consulted for further details [23,39,40,42].

The gelling temperature of a carrageenan solution is a function of the concentration and type of gelling cations present in the system. When removing the gelation-inducing cations from the medium, as well as from the polysaccharide, the obtained polymeric solution does not form a gel irrespective of the applied temperature. As long as gelling cations are present, the carrageenan solution will gel at a specific temperature. In fact, the higher the cation concentration, the greater is the gelling temperature [42]. The gelling temperature of κ-carrageenan ranges from 35 to 65 °C. *Iota* type carrageenan has a higher gelling temperature than κ-carrageenan at the same equivalent concentration of their respective strongest gelling cations [41].

Apart from the various types of carrageenan, the literature also describes several hybrid carrageenans. The term hybrid refers to the co-occurrence of different disaccharide units in the polymer, which are found in native or unprocessed κ- and ι-carrageenan chains [43]. However, these molecules have been used as gelling agents mainly in applications of food industry [44,45,46,47], thus being out of the scope of this review.

### 2.4. Biological Activity

Apart from the physicochemical properties, which justify most of the interest on this carbohydrate, the inherent biological activities of the polymer have also increased its biomedical interest. In this regard, several biological activities have been reported for carrageenan over the last years, mainly including anticoagulant, antiviral and antitumor activities [8]. Based on this, carrageenans have been tested in therapeutic approaches of respiratory weaknesses, ranging from the common cold [48] to the infection with influenza virus H1N1 [49]. Other viral infections, such as hepatitis A, herpes and dengue [50] have also been addressed. Other studies have shown the antitumor and immunomodulation activities of carrageenan [51,52], as well as anticoagulant properties [53,54]. In addition, cholesterol- and lipid-lowering effects of carrageenan have been demonstrated in a clinical trial, showing that carrageenan can significantly reduce serum cholesterol and triglyceride levels [55]. Furthermore, carrageenan is well known for its inflammatory capacity, being inclusively used in one of the most frequent protocols for inflammation induction regarding the assessment of anti-inflammatory substances [25]. A very complete review on carrageenan biological activities has been recently published [56] and these activities were also reviewed in [25].

## 3. Fucoidan: Sulfated Polysaccharide of Brown Seaweeds

Fucoidan, first isolated by Kylin in 1913, designates a family of sulfated polysaccharides extracted from marine brown algae (Phaeophycophyta) and some echinoderms (sea urchin and sea cucumber). It was first called “fucoidin”, but it is now named fucoidan, according to the International Union of Pure and Applied Chemistry (IUPAC) recommendations. The term fucoidan is commonly applied for complex sulfated polysaccharides, often isolated from marine algae, mainly containing fucose residues, but also many other monosaccharides. In turn, the term sulfated fucan is reserved for polysaccharides with a regular structure, containing a majority of sulfated fucose, which are often extracted from marine invertebrates such as sea cucumber and sea urchin. However, not all authors consider these denominations and, therefore, the polymer is usually indistinctly termed fucoidan or fucan, while other terms like fucosan might also be referred [57,58].

Over the last years, there has been a growing interest among producers and consumers in using new functional ingredients in the diets, due to various beneficial health effects. In this context, research from the past decade has provided extensive scientific evidence on the health benefits of fucoidan. The biological properties of this polymer (to be mentioned later on in the section) have supported its application as functional food for disease prevention and health promotion [59]. Besides, fucoidan has revealed potential applications in nutraceutical, cosmeceutical and pharmaceutical industries as well [60], the latter counting with several applications in therapeutic approaches [61]. However, determining how this active polysaccharide may retain its functional properties in different processing steps of the particular industrial applications remains to be demonstrated and studied [60].

### 3.1. Origin, Extraction and Processing

Brown algae (Phaeophyta), the second most abundant group of algae, produce a range of active components. The structure of their cell walls consists of an amorphous matrix of acid polysaccharides, linked to each other by proteins. These acid polysaccharides are mainly composed of fucoidan and alginic acid, which confer structural toughness and flexibility to seaweed [60]. Fucoidan usually constitutes about 5%–10% of dry algal biomass, depending on the species, the part of the thallus being used and the harvesting period [62]. In the recent years, these fucose-containing polysaccharides have been isolated from different sources (see Table 1). Before choosing the source, it is important to consider that fucoidan can differ in structure among algal species and may even vary within the same species. Because of the heterogeneity of fucoidan structures, different extraction conditions may lead to the isolation of distinct fucoidan forms [38].

Isolation and purification of fucoidan from marine algae are generally carried out through the following steps: collecting, washing, drying and milling of the raw material; pretreatment of algae; fucoidan extraction with extracting agents such as hot water, dilute acid or alkali; isolation and purification of fucoidan by fractional precipitation with ethanol [63], lead salts [64], calcium salts [65], quaternary ammonium salts [66] or by the use of anion exchanger columns [67]; and finally freeze-drying of fucoidan extracts. It must be considered that some extraction methods may alter the natural structure and destroy the sulfation pattern and, thus, the bioactivity and physicochemical properties of fucoidan may be affected [68].

### 3.2. Chemical Structure

Although fucoidan has been known for over a century, its chemical structure is still incompletely determined, owing to its heterogeneity and irregularity. This is due to the fact that marine brown algae synthesize highly branched polysaccharides, which structures and proportion vary in dependence of the specific taxonomic position. For instance, it has been shown that fucoidan obtained from representatives of Chordariales and Laminariales may display different backbone structure compared with that isolated from algae belonging to the order Fucales [58,67,69]. In addition, more than one type of fucoidan may occur simultaneously in the same algal species [70]. The major sulfated polysaccharide of brown seaweed differs from red algae polysaccharides in the main sugar backbone which is galactose for carrageenan and fucose for fucoidan [64]. In fact, fucoidan essentially consists of α-l-fucose units (usually referred as α-l-fucopyranose). Sulfation of α-l-fucose residues may occur at positions C-2 and/or C-4 and, though it is rare, also at position C-3; but the structure and sulfation pattern of the sugar-backbone are species-related [71,72].

**Figure 4:**
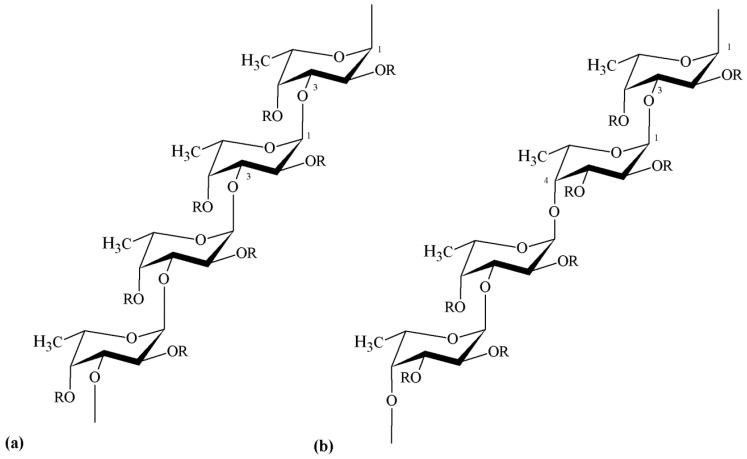
Scheme of α-l-fucose chains observed in fucoidans isolated from several algae belonging to the taxonomic orders Chordariales and Laminariales (**a**) and Fucales (**b**); (**a**) The chain is only composed of repeating (1→3)-linked α-l-fucose residues; (**b**) The chain consists of alternating (1→3)- and (1→4)-linked α-l-fucose residues. *R* represents the positions of potential attachment of carbohydrate residues (glucuronic acid, mannose, galactose, xylose, α-l-fucose, fucoside) and non-carbohydrate (sulfate and acetate) substituents.

Besides fucose and sulfate, fucoidan may also contain additional sugar constituents, including mannose, galactose, glucose, xylose, uronic acids and yet acetyl groups [14,65,73,74]. The presence of these additional components, sometimes in appreciable amounts, has not yet been established as a regular phenomenon. On the other hand, a certain similarity in the backbone structure of different fucoidan molecules has been observed, regarding the positions of inter-glycosidic linkages. Many studies show that several representatives of the orders Chordariales and Laminariales contain fucoidan with a linear backbone composed of (1→3)-linked α-l-fucose residues. However, fucoidan isolated from algae belonging to the order Fucales mostly display a backbone composed of alternating (1→3)- and (1→4)-linked α-l-fucose residues [66,72,73,75]. Representative backbone structures of these fucoidans are depicted in Figure 4.

These findings may not be considered a pattern, though. For instance, fucoidan isolated from brown seaweed species of the order Fucales have also been reported to have fucose and galactose in comparable amounts; these structures are generally referred to as sulfated galactofucans. These are mainly composed of (1→6)-β-d-galactose and/or (1→2)-β-d-mannose units [14]. In these cases, fucoidan molecules not only differ in composition, but also in terms of glycosidic bond positions. In general, fucoidan polysaccharides may be branched, presenting a variety of substituting groups and side chain compositions. The typical positions referred to link sulfate groups (C-2, C-3 and/or C-4) may also be occupied by acetate groups (e.g., *Saccharina latissima*, *Chorda filum*, *Fucus* sp.). Neutral and partially sulfated residues of glucuronic acid, mannose, galactose and xylose have been further reported as side chain (e.g., *Sargassum* sp., *Fucus serratus*, *Punctaria plantaginea*, *Ascophyllum nodosum*). More complex, single fucosyl residue and fucoside (oligosaccharide of fucose) may also constitute the side chain of fucoidans (ex. *Cladosiphon okamuranus*, *Chorda filum*) [57,66,72,73,76,77].

Nevertheless, the reported structural data for fucoidan polysaccharide isolated from different brown seaweed species clearly indicate that there is no consistent basic structure of this polymer. The investment observed in recent years in research dedicated to obtaining highly purified fractions of the polysaccharide, is expected to permit a better understanding of fucoidan structures [78,79,80].

### 3.3. Physicochemical Properties

As mentioned above, the physicochemical and biological properties of algal polysaccharides are strongly correlated to their chemical composition. In this regard, an interesting review provides an in-depth approach to the relations between the structure, functions and metabolic paths of fucoidan [81]. The chemical composition of seaweeds, in turn, is the result of many factors such as harvesting region, season and the specific algal species [82]. All these aspects naturally result in a complex and variable chemical structure of algal fucoidans, which reflects the differences in biosynthesis. These structural irregularities, including the presence of numerous minor sugar and non-sugar compounds, random sulfation and/or acetylation, make structural analysis of these polysaccharides a difficult task [62,83]. Nevertheless, some studies have shown examples of regularity in the structure of fucoidans [75,84].

The first works on chemical composition of brown seaweed polysaccharides focused on fucoidans. They have been classified, according to their chemical composition, as ascophyllans, glycuronofuco-galactans sulfate and fucoidans (homofucans). The latter has the simplest chemical structure, *i.e.*, homofucans are polysaccharides consisting of sulfated fucose only [85]. However, the term “fucoidan” is commonly used to describe the other fucose-containing heteropolysaccharides.

In addition, structural modifications, such as desulfation, oversulfation, acetylation and benzoylation, allow the development of derivatives of fucoidans. In general, the natural sulfation grade ranges between 4% and 8%, depending on the site and season of collection of the algae [86]. Fucoidan has been demonstrated to bind to a large number of compounds, including proteins. The binding affinity appears to be mainly determined by the negative charge of the polymer, the molecular weight and degree of sulfation, rather than by any specific structure of the carbohydrate [87,88,89].

Fucoidans are not only highly heterogeneous polysaccharides regarding the sugar composition and sulfate content, but also concerning their molecular weight. This may vary from 10 [89] to approximately 2000 kDa [61]. Lower molecular weight fucoidans can be prepared by chemical, physical or enzymatic means to obtain oligosaccharides with more diverse bioactivities [38]. Acidic hydrolysis of fucoidan leads to sulfated fucoses and oligosaccharide fragments [80], which may be also obtained by autohydrolysis [90,91], by a radical process involving a hydrogen peroxide-cupric redox system [92] or by enzymatic cleavage [69,93]. A disadvantage of the chemical hydrolysis is that it is quite unspecific. Additionally, high acid concentrations may destroy the sulfation pattern and the polysaccharide chain, which may lead to inactive monosaccharides [58]. Oppositely, enzymatic modifications of fucoidans can be done by a group of hydrolases, the so called fucoidanases or α-l-fucosidases. These are able to specifically cleave glycosidic bonds in the polysaccharide chain, while preserving the sulfation pattern and, thus, the basic physicochemical properties of fucoidan [68].

Knowledge on the solubility and rheological properties of fucoidan is important to understand and establish different applications. Fucoidan is very soluble once extracted and the solubility is related to the level of branching, depending on the content of sulfate groups. There are, however, very few reports in the literature on the rheological characteristics of fucoidan isolated from brown seaweeds [94,95,96]. Despite of its hygroscopic behavior, fucoidan does not develop highly viscous solutions [85], so the polymer is not industrially used as thickening or gelling agent, as many other polysaccharides. In fact, a study showed that partially purified fucoidan from *Laminaria religiosa*, *Undaria pinnatifida*, *Hizikia fusiforme* and *Sargassum fulvellum* produced aqueous solutions of low apparent viscosity with pseudoplastic flow behavior [94]. Complementarily, it was reported that fucoidan from *F. vesiculosus* exhibited Newtonian behavior and had the highest viscosity, when compared to the species *Saccharina longicruris* and *Ascophyllum nodosum* [97]. Moreover, it was reported that the dynamic viscoelasticity of fucoidan isolated from commercially cultured *Cladosiphom okamuranus* increased linearly with an increase of fucoidan concentration up to 2% (*w*/*w*) and decreased gradually with increase in temperature. Additionally, fucoidan viscoelasticity increases with addition of NaCl, CaCl_2_ and sugar [94,95]. Besides, the dynamic viscoelasticity of the polymer was stable over a wide pH range (5.8 to 9.5), indicating that fucoidan molecules are stable under acidic and alkaline conditions [95,98]. In this perspective, it seems that the viscosity of fucoidan is influenced by algae species, concentration, molecular weight, presence of sulfate groups, branching, pH and temperature. More extensive research is however needed to enable the establishment of a straight relationship between viscosity and fucoidan structure [97].

In contrast to carrageenan and ulvan (the latter being another sulfated polysaccharide to be described in the next section), there is little evidence about gelling and film forming properties of fucoidan [83]. In fact, gelation of fucoidan was not observed up to 25% concentration [41]. It has been reported, however, that upon mixing with other polymers, particularly those of opposite net charge, the formation of gels and films is enabled based on electrostatic interactions between negatively charged sulfate groups of fucoidan and positively charged groups of the other polymers. Examples of these structures have been evidenced with chitosan and poly(2-hydroxyethyl methacrylate) [99,100].

### 3.4. Biological Activity

As mentioned for carrageenan, fucoidan and its oligosaccharides have been extensively studied regarding the evidence of diverse biological activities. These include antitumor effect [101], antiviral [102], anticoagulant [89] and anti-inflammatory activities [103]. Based on the reports, these properties are related to molecular size, type of sugar content, sulfation degree and molecular structure. From all the reported biological activities, the potent anticoagulant property of fucoidan is by far the most widely investigated [104,105,106]. Many studies showed that this anticoagulant activity is possibly related to the sulfate content and the position of sulfate groups, molecular weight and sugar composition. Furthermore, fucoidan requires an enough long sugar-chain and a certain conformation to bind to thrombin, so apparently a relatively large molecular weight is needed to achieve anticoagulant activity. However, it was also demonstrated that branched structures are not always necessary for an anticoagulant action [57].

Additionally, it has been shown that the carbohydrate further has potent antiviral effect against herpes simplex virus type 1 (HSV-1), HSV-2 and human cytomegalovirus [102]. Yet, the anti-metastasis and anti-lymphangiogenesis activities of fucoidan, as well as its immunomodulatory effect, have been demonstrated [107,108].

The biological activities of fucoidan were recently reviewed, with a particular focus on antitumor activity [109]. For all these properties, fucoidan has been finding applications in the biopharmaceutical industry [60] and, in the recent years, the interest in this sulfated carbohydrate has also been extended to biomedical-related fields, including tissue engineering [6].

## 4. Ulvan: Sulfated Polysaccharide of Green Seaweeds

Classification of algae has not always been an easy task and, specifically regarding green algae, the complexity increased when recent genetic studies revealed, for instance, that the green seaweeds *Enteromorpha* and *Ulva* are not of distinct genera [110]. Despite of the fact that researchers extract biopolymers from different genus and species of green algae using many distinct extraction methods, it is now generally accepted that ulvan designates a group of sulfated polysaccharides extracted from green seaweed. A relevant aspect to highlight is the fact that, from the sulfated polysaccharides presented in this review, ulvan is by far the less studied.

Green marine algae are distributed worldwide and considered an important food source in many parts of the world as a marine vegetable. *Ulva* spp. (commonly known as sea lettuce) is a rich natural source of carbohydrates, vitamins, essential amino acids, minerals and dietary fibers [111,112]. Ulvan is currently receiving a great deal of attention, owing to physicochemical and biological properties of potential interest for agriculture [113] and pharmaceutical applications [114,115,116,117]. These properties are highly dependent on the chemical composition, charge density and molecular weight of ulvan [118], as also referred for the other polysaccharides. Furthermore, as observed in other seaweed divisions, the yield and specific composition of polysaccharides from green algae depend on environmental factors, such as the species from which they are obtained, [20] the season of collection [119] and the employed extraction method [120].

### 4.1. Origin, Extraction and Processing

After its first identification in the early 1940s–1950s, researchers have been struggling with the processing and characterization of ulvan. As described for carrageenan and fucoidan, the overall procedure to obtain ulvan from green algae initiates with selection, collection and identification of the raw material. This step is followed by algae stabilization and grinding. The stabilization can be performed by several alternative procedures, including freezing, drying methods, brining and dry salting, a selection that has a considerable impact on the final yield of extraction [120].

Ulvan extraction is mostly performed with hot water solutions [121,122] and might be further improved by the presence of calcium chelating agents [118], acidic or alkaline solutions [123]. The purification of the polymer to eliminate pigments, lipids, amino acids and peptides has been reported using various procedures [115,119,123,124,125,126,127,128] and organic solvents [22,121,123,129]. Generally, a polysaccharide with improved purity is obtained by precipitation with organic solvents, frequently ethanol [22,115,126]. Finally, ulvan aqueous extract can be concentrated in a rotary evaporator [125] or dried by freeze-drying or hot air-drying [129,130]. The removal of impurities, as well as the drying of ulvan extract, may favor the modification of the polysaccharide conformation and properties [131]. In fact, a study reported the effects of time and temperature on ulvan degradation, indicating that temperature was the main factor affecting the rate of depolymerization [21]. Besides, the use of different solvents to extract ulvan will result in extracts with varying composition and, thus, different biological and physicochemical properties [120].

### 4.2. Chemical Structure

**Figure 5:**
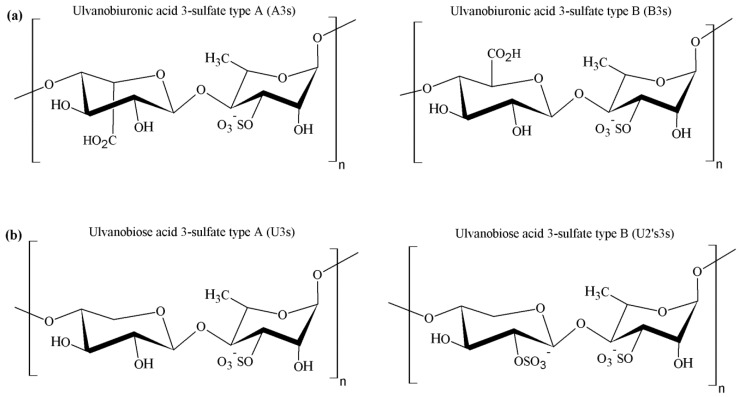
Structure of the main repeating disaccharides in ulvan isolated from *Ulva* sp. (**a**) Ulvanobiuronic acid type A3s disaccharide is composed of glucuronic acid and sulfated rhamnose, whereas type B3s consists of iduronic acid and sulfated rhamnose; (**b**) Ulvanobiose acids in which xylose or sulfated xylose residues occur in place of uronic acids.

Ulvan corresponds to the major biopolymeric fraction isolated from green seaweed cell walls, showing a structure of great complexity and variability [123]. The pioneering works from Brading *et al.* [123] and Percival *et al.* [121] established that sulfate, rhamnose, xylose and glucuronic acid are the main constituents of ulvan, showing the structure depicted in Figure 5. However, it was only after the work of Quemener *et al.* [128] that iduronic acid was recognized as a constituent carbohydrate unit in ulvan.

The major repeating disaccharide in the ulvan extracted from different ulva samples was found to comprise two different types of aldobiouronic acid. These were named ulvanobiuronic acid 3-sulfate type A and type B (A3s and B3s, respectively). The A3s disaccharide is composed of glucuronic acid and sulfated rhamnose, while type B3s consists of iduronic acid and sulfated rhamnose, mainly associated via (1→4) glycosidic linkages. Rhamnose residues are sulfated mainly at position C-3 or at both positions C-2 and C-3. In some ulva extracts, xylose or sulfated xylose residues may occur in place of uronic acids, as shown in Figure 4. In this case, the disaccharides are called ulvanobiose acids and symbolized as U3s (ulvanobiose acid 3-sulfate) and U2’s3s (ulvanobiose acid 2,3-disulfate). Low proportions of galactose, glucose and mannose have been reported, but their real integration in ulvan structure has been questioned [20,132,133,134].

### 4.3. Physicochemical Properties

Generally, ulvan exhibits some of the hydroxyl groups of the sugar residues substituted by sulfate groups (Figure 4). As explained before, these biopolymers are constituted by complex highly branched molecules which do not appear to have a defined backbone or simple repeating unit. Nor do they appear to have long chains of a single sugar [1]. The sugar composition of ulvans is extremely variable, being the most frequent rhamnose (16.8%–45.0%), xylose (2.1%–12.0%), glucose (0.5%–6.4%), glucuronic acid (6.5%–19.0%) and iduronic acid (1.1%–9.1%). Mannose, galactose and arabinose have also been found in ulvan from some *Ulva* species. Determining the sugar sequence in ulvan thus represents a major challenge. Oligosaccharides and oxidation products released after mild acid hydrolysis of native and chemically modified ulvan suggested the presence of rhamnose, xylose, glucuronic acid or glucose, all present in the same chain. Moreover, it was also indicated that glucuronic acid can occur as branches on C-2 of rhamnose [20]. Anyhow, the polysaccharide composition may be even more complex and is known to be influenced by seaweed species, algal seasonality and the mode of preservation of algae [135], as is usually described for many algal polysaccharides.

The heterogeneous chemical composition of ulvan leads to an essentially disordered conformation of the biopolymer. In spite of this disordered structure, the local regularity given by the repeating aldobiouronic units, for instance, is believed to be sufficient for the formation of transient “junction zones” responsible for the formation of the weak gel that ulvan is known to produce in native state [127].

Many characteristics and properties of ulvan remain unknown when the extraction conditions vary, namely the rheological and textural properties. According to the literature, there are few reports on the rheological properties of ulvan polysaccharides extracted under particular conditions. However, the impact of extraction procedures on the chemical, textural and rheological properties of ulvan extracts from *Ulva lactuca* was recently assessed. Regarding rheological characteristics, results demonstrated a great contribution of the extraction method on these properties. Ulvan extracts have generally demonstrated a pseudoplastic behavior, a viscosity decrease being observed as the shear rate increased [136]. Furthermore, as observed for carrageenan, ulvan has been shown to produce viscous solutions when dissolved in water. It also forms gels in presence of B^+^ and Ca^2+^ ions at basic pH by yet an unclear mechanism; and the gelling ability was shown to depend on the presence of divalent cations [137,138]. Lahaye and Axelos [130] studied the influence of pH, buffer type and the amount of added ions (Ca^2+^ and B^+^) on the gelling characteristics of ulvan extracted with boiling water after enzymatic treatment of algae. In a subsequent work, the thermo-reversibility of the gel formed by ulvan from *Ulva rigida*, during heating and cooling was studied. According to the authors, ulvan yields viscous aqueous solutions that can form thermo-reversible gels in the presence of Ca^2+^ and B^+^ ions at basic pH. It was also shown that gel formation is a time-dependent process and the viscoelastic behavior of the ulvan solution in the presence of ions (pH 7.5) indicated that the investigated extracts led to systems with properties close to a solid, rather than liquid material. Other authors showed the impact of stabilization treatments (freezing, freeze-drying, hot-air drying, brining and dry salting) of *Ulva rotundata* on the physicochemical and rheological properties of ulvan that was extracted using oxalate sodium, followed by water extraction [120]. As a whole, these studies revealed that ulvan gels are thermo-reversible and that high or low ion concentration, as well as pH variations, may influence ulvan conformation, and, thus, the gel formation.

The various forms of ulvan markedly differ in the intrinsic viscosity and molecular weight. Regarding the latter, different molecular weights and molecular weight distributions have been reported. Sedimentation measurements indicated molecular weights ranging from 530 kDa to 3.6 × 10^3^ kDa for ulvans obtained from *U. pertusa*, *U. conglobata* and *E. prolifera*. Great variations were found in ulvans extracted from *U. conglobata* depending on the temperature at which the extraction was performed. This indicates that different molecular weight ulvans can be obtained by changing the temperature of extraction, a high temperature being required to extract high molecular weight ulvan [20]. It is worth noting that ulvan extracts with lower molecular weights (28.2–151.7 kDa ) can be obtained by using H_2_O_2_ treatment [21].

Ulvan is considered to have a high charge density, which determines its water-solubility. However, it has a certain hydrophobic character, possibly determined by the presence of a great amount of methyl groups in the rhamnose repeating unit. Notwithstanding the aqueous solubility of the polysaccharide, a study performing ultrastructural analysis revealed the presence of spherical shaped aggregates of ulvan in aqueous solution. As a polyelectrolyte, both the ionic strength and the pH of the used solvent play a role on the solubility and morphology of ulvan, since the type and amount of counterions in solution could contribute to the condensation of the polymer [131]. Therefore, it is very important to bear in mind that using this polymer, as any other with polyelectrolyte character, implies a good optimization of conditions regarding the final objective of its application.

### 4.4. Biological Activity

As for the previously reviewed sulfated polysaccharides, different bioactivities have been attributed to ulvan. The polysaccharide has been demonstrating to have antioxidant activity, which is apparently dependent on molecular weight, since low molecular weight ulvan shows stronger antioxidant activity compared to larger fractions [21]. Antilipidemic effect has also been registered. In this regard, ulvan has been reported to reduce total serum cholesterol, triglycerides and low density lipoprotein (LDL) cholesterol, while elevating high density lipoprotein (HDL) cholesterol levels. This effect was identified to depend on the molecular weight of ulvan fractions, as high molecular weight fraction is more effective on total serum and LDL-cholesterol, whereas low molecular weight fractions are more effective on triglycerides and HDL-cholesterol [114,139].

Ulvan has also been studied for antiviral activity *in vitro* against a number of human and avian influenza viruses. In fact, Ivanova *et al.* [140] described that ulvan polysaccharides isolated from green algae had good inhibitory effect on influenza A virus, the inhibition effect being dose-dependent and strain-specific. Likewise, ulvan has been shown to have high and specific activity against herpes simplex virus [141].

As for carrageenan and fucoidan, the reported biological activities depend on sugar composition, molecular weight and sulfate content of ulvan and thus, as above-mentioned, on genus, species and ecological and environmental factors. Anyhow, an attractive use and exploitation of green algae would take advantage of these biological properties and apply them in pharmaceutical and biomedical fields. However, among the three main divisions of macroalgae, green algae remain a rather underexploited biomass, particularly in areas where other sulfated polysaccharides of algal origin have already proven their value [6].

## 5. Drug Delivery Systems Based on Seaweed Sulfated Polysaccharides

Drug substances are usually not administered as they are in pure state, but rather as part of a dosage form where they are frequently combined with other agents (excipients). In many occasions, excipients act as simple inert supports of the active molecule(s) [142], but it is also true that multifunctional excipients have been being increasingly used. The pharmaceutical industry uses excipients from a wide variety of sources, both synthetic and natural. Among those of natural origin, polysaccharide-based excipients have been registering increased application, because of their ability to produce a wide range of materials, carriers and devices, owing to specific properties, including molecular weights. Besides, one of the properties most often referred for polysaccharides is their structural flexibility. This feature enables the chemical modification of the polymers to fulfill the requirements of specific drug delivery systems, thus allowing a direct competition with the synthetic excipients available in the market [143]. The enormous orientation of pharmaceutical industry towards naturally derived polymers has become a subject of increasing interest, driving the continuous exploitation of such compounds [142]. Furthermore, the biomedical field, including tissue engineering, regenerative medicine and drug delivery, is constantly looking for new biomaterials with innovative properties. Polysaccharides are potential candidates, not only because of their propensity for biocompatibility and biodegradability, but also due to their high availability at relatively low cost. In this context, sulfated polysaccharides present in different marine algae species have been gathering great interest, which is a reflex of the continuous growing of knowledge on chemical and biological activities of these compounds [6].

The specific application of polysaccharides in pharmaceutical formulations include their use in the manufacture of solid monolithic matrix systems, implants, films, beads, microparticles, nanoparticles, inhalable and injectable systems, as well as hydrogel formulations. It happens frequently that carriers like nanoparticles, microparticles and beads prepared with polymers having gelling ability are called hydrogels. However, for the effects of this review, all carriers exhibiting a particulate morphology/structure were treated as such, independently of the terminology originally used in the primary references. Within the dosage forms listed above, the carbohydrate polymers might serve rather different functions, including the use as binders, coatings, matrix materials, drug release modifiers, thickeners, stabilizers, disintegrants, solubilizers, emulsifiers, suspending agents, gelling agents and bioadhesives [144]. Occasionally, some of the referred functions are cumulative.

As mentioned before, the emphasis of this review is placed on the three algae-derived sulfated polysaccharides carrageenan, fucoidan and ulvan. Owing to their particular features, described in detail in the previous section, a growing interest is being observed regarding a biopharmaceutical application in drug delivery. Particularly, carrageenan-based pellets [145,146,147], beads [148,149,150,151,152,153,154,155,156,157], nanoparticles [158,159,160,161,162,163,164,165,166,167], microparticles [168,169,170,171,172,173,174,175], hydrogels [176,177,178,179,180,181,182,183,184,185], films [186,187,188,189,190,191,192,193], matrices [194,195] and other devices [196,197,198,199,200] have been extensively investigated as drug delivery carriers. In turn, the research on fucoidan for this purpose has also increased in recent years. Fucoidan-based drug carriers, such as nanoparticles [201,202,203,204,205,206,207,208,209,210,211,212], microparticles [213,214,215,216,217] and hydrogels [99,218] have also been successfully developed. Differently, ulvan remains a rather unexploited biomaterial for an application in the design of drug delivery systems. Despite its chemical and biological versatility, very few studies on ulvan biomedical applications have been reported to date [133,134,219,220], although some address drug delivery approaches [221,222,223].

There are two major elements contributing for the importance and relevance of biomaterials based on sulfated polysaccharides with application in pharmaceutical biotechnology: (1) the glycosidic bonds, which can be easily cleaved by hydrolase enzymes and, thus, contribute for biodegradability; and (2) the presence of the negatively charged sulfate groups that potentiate polyelectrolyte behavior and permit functionalization for specific applications [224], apart from a privileged interaction with negatively charged epithelia. Additionally, the presence of hydroxyl groups (OH) on the structure of these polymers provides the necessary moieties for several chemical modifications. In this regard, the introduction of hydrophobic, acidic or basic groups, or even other functionalities into polysaccharide structures might alter the properties of biopolymers, enabling specific tailoring towards the devised objectives. Taking benefit from these features, the use of these carbohydrates in drug delivery applications has been proposed frequently. From the three, carrageenan is by far the most reported, as indicated in Figure 1, which is certainly a result of its first isolation, easy purification and well-defined chemical structure.

For an easier structuration of the review, the application of the polysaccharides in the design of different carriers is arranged and presented according to the carrier-types.

### 5.1. Nano and Microparticles

Particulate carriers have been developed as a physical approach to alter and improve the pharmacokinetic and pharmacodynamic properties of various types of drug molecules. Compared with conventional dosage forms, particulate delivery systems offer many advantages, such as availability for delivery through various routes of administration, tailoring of particle size and surface characteristics and, in some cases, possibility to offer controlled and sustained release of the drug at specific sites [225,226].

Nano- and microparticles are the most referred of the particulate carriers, in the majority of cases presenting a matrix composed by polymeric materials. Although the definition may not be consensual under all instances, nanoparticle is the term frequently used for spherical particles with diameters ranging from 10 to 1000 nm, whereas microparticles present diameters in the micrometer range (typically from 1 μm to 1000 μm). Drugs can be dissolved, entrapped, encapsulated or attached to the polymer matrix of the particulate carriers. Structurally, these systems are divided in two categories: nanocapsules/microcapsules in which the drug is mainly confined to a cavity surrounded by a polymer membrane (shell); and nanospheres/microspheres in which the drug is dispersed within the polymeric matrix, according to a classification that is now widely accepted [227].

Sulfated seaweed polysaccharides have been finding applications in the production of nanoparticles and microparticles, mainly owing to their ionic nature. This enables the formation of complexes with oppositely charged polyelectrolytes, which has been found very useful regarding the design of drug carriers, since polyelectrolyte complexes allow the association of drugs in the polymer matrix at a molecular level. Such structures permit drug entrapment during precipitation of the complex, or through absorption to the already formed complexes. The drug can also be chemically bound to one of the polymers and be incorporated during the complexation. Afterwards, the drug is released from the polyelectrolyte complex either by ion exchange mechanism or by charge interaction, as well as by polymer breakdown and dissolution of the complex [228].

#### 5.1.1. Nanoparticles

Polymeric nanoparticles have been extensively studied for drug delivery purposes and varied methods have been developed for their production, including emulsification, coacervation, ionic gelation and polyelectrolyte complexation, among others. All these methods comprise bottom-up fabrication processes, which involve the assembly of molecules in solution to form defined structures, in this case, nanoparticles. Readers interested in a detailed analysis of these methodological approaches are directed to the reviews [229,230].

**Figure 6:**
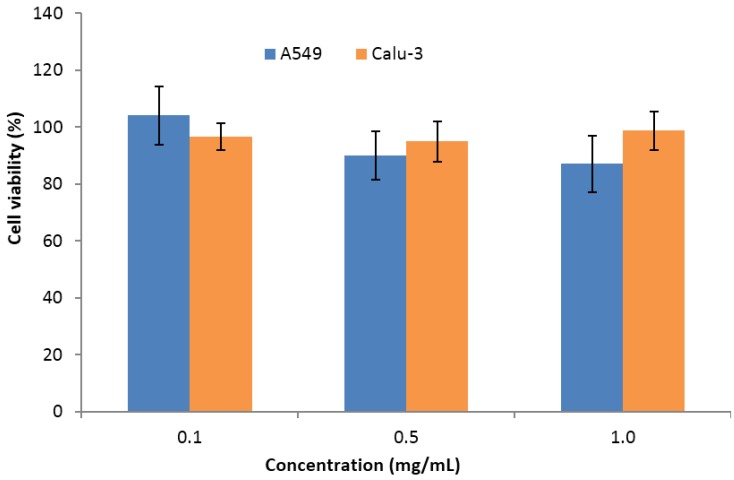
Calu-3 and A549 cell viability measured by the methyltetrazolium (MTT) assay after 24 h of exposure to chitosan/carrageenan nanoparticles. Data represent mean ± SEM (*n* = 6). Adapted with permission from [163].

The use of sulfated polysaccharides has been explored in the design of polymeric nanoparticles, mainly taking advantage of the sulfate content, which directly results in the exhibited negative charge. The complexation with cationic polymers is, thus, frequently used as the driving force towards the formation of nano-sized carriers. In this regard, carrageenan has been mostly referred to be complexed with chitosan [160,161,162,163,169,231,232,233,234,235,236], but the use of other counterions such as protamine [237] and a cationized pullulan [162], or a direct complexation with drugs [238] were also reported. A recent work reviewed the application of carrageenan in drug delivery, including the production of nanoparticles [9] and, therefore, we will focus on works not comprised in that review or which are considered relevant. Our group was one of the first to report chitosan/κ-carrageenan nanoparticles [160,161,163], testing cross-linkers, demonstrating the ability to encapsulate proteins, evaluating the storage stability and the stability in presence of lysozyme and assessing the cytotoxicity of the complexes. Regarding the latter, a methyltetrazolium (MTT) assay performed in two respiratory cell lines (A549 and Calu-3) revealed an absence of toxicity in concentrations up to 1 mg/mL exposed for 24 h (Figure 6). Additionally, a strategy was proposed for the delivery of the nanoparticles by inhalation, mediated by microparticles-containing-nanoparticles [163].

The effect of different types of carrageenan (κ-, ι-, λ-) and varied polymeric charge ratios on the final characteristics of chitosan/carrageenan nanoparticles was explored by other authors. Nanoparticles formulated with κ-carrageenan were those showing the higher encapsulation efficiencies (up to 79%) of glucose oxidase, used as model molecule, while the charge ratio also played a role in the association capacity. A controlled release was observed when the nanoparticles were treated with different physiological and enzyme solutions; κ-carrageenan/chitosan nanoparticles being those showing the lowest release rate [164]. A controlled release was also reported for erythropoietin (48% encapsulation efficiency), which released 50% over a two week period [233]. Notwithstanding the predomination of polyelectrolyte complexation, other methodological approaches have provided the incorporation of carrageenan in nanoparticulates, mainly in the form of coatings [158,165,166]. Reverse microemulsion combined with thermally induced gelation was also reported [159]. As a whole, carrageenan-based nanoparticles have been proposed for the encapsulation and delivery of a wide variety of molecules, including proteins [161,162,163], antibiotics [167,238], DNA [239] and anticancer drugs [165].

**Figure 7:**
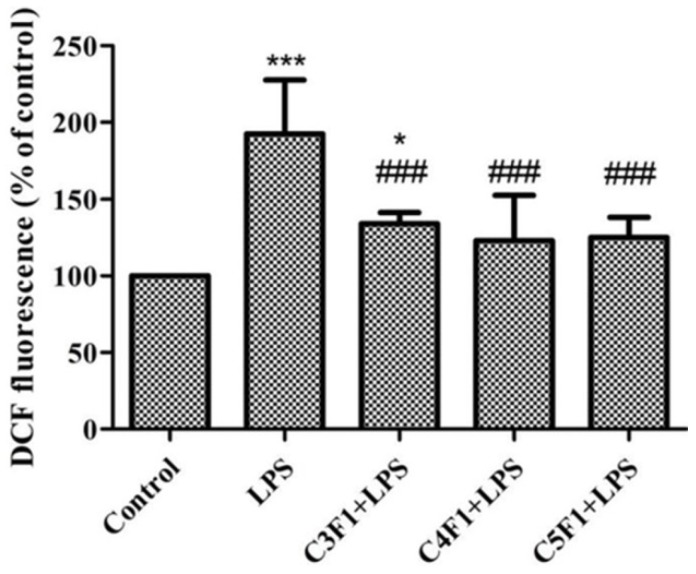
Lipopolysaccharide-induced RAW 264.7 cells detected by flow cytometry. Data are mean ± SD of values calculated on 5 distinct batches (*n* = 5). Statistical analysis was performed by one-way ANOVA. * *p* < 0.01 *versus* control. *** *p* < 0.001 *versus* control. ### *p* < 0.001 *versus* LPS. Adapted with permission from [207].

Fucoidan has also been used as matrix material in nanoparticle development, although to a lower extent when comparing with carrageenan. Many nanoparticle formulations have been proposed in recent years for biomedical applications. Again, polyelectrolyte complexation was the predominant technique, enabled by the presence of the sulfate groups, and chitosan was used in the majority of cases as counterion [201,202,203,204,206,207,208,209,240]. The polymeric interactions taking place during nanoparticle formation were exhaustively characterized in recent works [205,241]. Interestingly, fucoidan/chitosan nanoparticles have been shown to exhibit a pH-sensitive behavior, demonstrating the sustained release of the antitumor drug curcumin in distinct pH buffer solutions that simulate the gastrointestinal environment. The sustained release of the drug occurred as the pH increased, especially when fucoidan/chitosan weight ratio was 1:1. The carriers were thus proposed for oral anticancer therapy [203]. The same route was envisaged for the delivery of berberine, an alkaloid with antimicrobial and anti-inflammatory activities, mediated by chitosan/fucoidan nanoparticles prepared with a fucoidan-taurine conjugate. Berberine was associated with efficiency around 40% and the nanoencapsulated form was suggested as a potential therapy for the treatment of diseases associated with intestinal epithelial dysfunction [202,206]. Yet another study suggested the loading of gentamicin in chitosan/fucoidan nanoparticles. Apart from efficiently associating (94%) and releasing the antibiotic (99% released in 72 h), the proper carriers further exhibited highly potent antioxidant effect. In fact, as depicted in Figure 7, the contact with nanoparticle formulations after exposure to lipopolysaccharide (LPS, a known generator of reactive oxygen species (ROS)) decreased the ROS level in macrophages (RAW 264.7 cells). The nanoparticles also scavenge 1,1-diphenyl-2-picrylhydrazyl (DPPH), an ability that was attributed by the authors to the fucoidan content [207].

**Figure 8:**
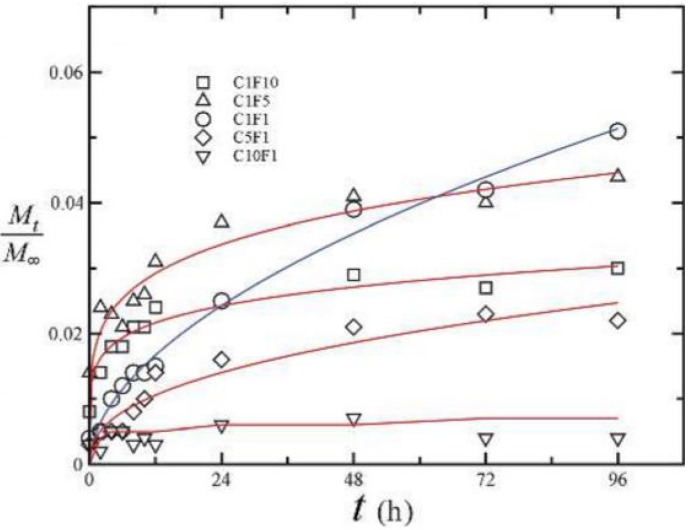
The release kinetics of basic fibroblast growth factor (bFGF) from chitosan/fucoidan nanoparticles, as measured by ELISA. Adapted with permission from [240].

The use as a carrier of basic fibroblast growth factor (bFGF) was also proposed in a nerve regeneration strategy, as bFGF has a marked positive effect on angiogenesis and neuronal cell survival. The nanoparticles provided a controlled release of bFGF, which was highly dependent on chitosan/fucoidan mass ratio. Curiously, as was reported above for curcumin, a higher control over the release rate was obtained for mass ratios of 1/1 (Figure 8), which showed a continuous release for four days. In turn, the other tested ratios tended for a plateau. This work also demonstrated that nanoparticles protect bFGF from heat and enzymatic deactivation, and decrease the amount of growth factor needed for neurite extension [240].

Still in the field of regenerative medicine, chitosan/fucoidan nanoparticles added of the cross-linker tripolyphosphate were described as adequate to deliver stromal cell-derived factor, which is an important chemokine in stem cells mobilization [208]. In a different approach, multilayer nanoparticles were successfully prepared through layer-by-layer assembly of fucoidan and chitosan, over a core of polystyrene. The fucoidan-chitosan particles showed ability for the encapsulation of poly-l-lysine, which was found to be released by a pH-dependent mechanism [209], further reinforcing the pH-sensitivity described in previous works.

One work was referred above describing a specific effect for the proper chitosan/fucoidan carriers, in that case a scavenging and antioxidant effect [207]. Interestingly, there are some other works on these nanosystems that do not envisage carrying a specific molecule, but instead explore the proper biological activities of fucoidan. In one such work, the known ability of fucoidan to inhibit angiogenesis was explored. An oversulfated fucoidan was synthesized to improve the anti-angiogenic effect and complexed with chitosan to produce nanoparticles intended for oral delivery. A more prolonged release of the oversulfated fucoidan was observed in simulated intestinal conditions comparing with gastric medium. Additionally, formulations with a positive surface charge provided the transient opening of Caco-2 cell tight junctions, an effect known to be featured by chitosan since a long time ago, improving the paracellular transport of fucoidan [210]. In another work, the nanoparticles were used to obtain an anticoagulant effect, which was two-fold higher than that provided by a fucoidan solution [242].

Other relevant works reported the preparation of fucoidan nanoparticles by other methods not involving the complexation with an oppositely charged molecule. An acetylated fucoidan formed nanoparticles containing doxorubicin by dialysis and the drug was shown to release according to first-order kinetics for 5 days. Macrophages treated with these fucoidan nanoparticles overexpressed various antitumor cytokines (tumor necrosis factor-alpha and granulocyte-macrophage colony-stimulating factor) [211]. Another work reported the grafting of fucoidan with hexadecylamine, which leads to self-assembled nanostructures that demonstrated anti-proliferative effect (inhibition of proliferation between 2% and 44%) in various tumor cells [212].

As reported above for carrageenan, in some cases fucoidan was used as coating of nanoparticles composed of other materials, with several objectives. The coating of iron nanoparticles was reported to improve the affinity by platelets, thus permitting the visualization of platelet-rich thrombus by magnetic resonance imaging [243], while that of poly(isobutylcyanoacrylate) nanoparticles improved cell uptake (macrophages and fibroblasts) [244]. In turn, the coating of pDNA/polyethyleneimine complexes was reported to reduce cytotoxicity towards B16-F10 cells (murine melanoma cells) [239]. As referred in Section 3.4, fucoidan has been reported to have antitumor effect and this feature has been explored in the design of fucoidan-based nanoparticles. In fact, there are several works reporting the cytotoxic effect of fucoidan nanoparticles, either when fucoidan is part of the nanoparticle matrix [211,212] or when it is used as coating material [245].

Considering its polyelectrolyte properties, ulvan may also establish ionic interactions with cationic compounds, being able to interact in this way with drugs and/or polymers, forming different complexes which have found application in the biomedical field [246,247]. Although some of the referred works consist in the development of ulvan-based biomaterials for tissue engineering, the application of this carbohydrate in the design of nano drug delivery systems is not described. As detailed in the previous section, this material has a structure that is still not perfectly defined and seems to be complex, thus certainly restraining the interest of researchers in investigating its applications.

#### 5.1.2. Microparticles

As stated before, polymeric microparticles including microspheres and microcapsules may be employed to deliver drugs in a rate-controlled and, sometimes, targeted manner. To date, ulvan has no reports in this regard, while carrageenan and fucoidan microparticles have found some applications. The use of carrageenan to prepare microparticles for drug delivery was reported in a limited number of occasions. To our knowledge, a first report appeared in 2000, when ι-carrageenan microparticles were proposed to encapsulate horseradish peroxidase, used as model protein. Microparticles formed by interaction with amines (mono-, di- or oligoamines), but the performed studies were no further than the association of the molecule and the evaluation of its stability [170]. Some years later, λ-carrageenan was combined with gelatin to obtain microspheres for ophthalmic delivery of timolol maleate, an anti-glaucoma drug. Different polymeric ratios proved to be useful in modulating drug release profile, a higher content of gelatin providing faster release. Yet, *in vivo* tests performed in rabbits revealed that the drug concentration and bioavailability in the aqueous humor were significantly high in comparison with commercial formulations [171]. The same group reported recently the ocular delivery of ciprofloxacin mediated by λ-carrageenan microparticles, but chondroitin sulfate microparticles revealed a better performance, comparatively [172]. Carrageenan microspheres were also reported to encapsulate allopurinol and local anesthetic agents, such as lidocaine hydrochloride, dibucaine hydrochloride and tetracaine hydrochloride. *Kappa*- and ι-carrageenan were used to prepare microparticles by spray-drying. The drug loading efficiency was over 70% and allopurinol and the local anesthetic agents released from the microspheres for at least 400–600 min, depending on the type of carrageenan [168]. The encapsulation of insulin regarding an oral therapy of diabetes has also been approached with κ-carrageenan microparticles, using a lectin functionalization. Insulin association efficiency was as high as 94% and the oral administration of insulin entrapped in the microparticles led to a prolonged duration of the hypoglycemic effect, up to 12–24 h, in diabetic rats [173]. A further study proposed the use of κ-carrageenan/calcium carbonate microparticles functionalized with folic acid to deliver doxorubicin to cancer cells. Highly porous microparticles were obtained which reduced cell viability in 87% in a human osteosarcoma cell line (MG-63) [175].

Regarding fucoidan, its application is much scarcer when compared with that of carrageenan. In fact, in the ambit of drug delivery the almost totality of carriers relies on the so-called fucospheres. These are microspheres obtained by polyelectrolyte complexation with chitosan and were first proposed for protein delivery. Bovine serum albumin (BSA) was used as model protein and encapsulated into the microspheres with efficiency up to 90%. The rate of drug release from the microspheres was dependent on the concentration of polymers involved in the particle production and also on the concentration of BSA [213]. Fucospheres were also shown to encapsulate and release ofloxacin, a broad-spectrum antibiotic [214] and a plasmid-encoded granulocyte colony-stimulating factor [215]. Furthermore, as also described for nanoparticles, in certain cases the polymeric carriers are used because of their proper characteristics, without encapsulating any drug. In this regard, fucospheres demonstrated *in vitro* and *in vivo* the ability to treat dermal burns, benefiting from an intrinsic capacity of fucoidan in tissue healing. Fucospheres shortened the treatment period of burns existing in rabbits, providing a fast and effective healing by improving regeneration and re-epithelization [99,216]. With this set of results, fucospheres were presented as a potential delivery system for different applications and for the encapsulation of different bioactive molecules.

Another work reported the preparation of fucoidan/chitosan microparticles using the coaxial electro-spray drying technique. Lipoic acid was used as model drug, releasing according to a profile comprising an initial burst until 4 h (50%–70%) and a more sustained release up to 48 h. Because of the design of the spray-drying apparatus, the microparticles were described to have a fucoidan core and a chitosan shell. The composite particles underwent physical gelation upon contact with water and showed a unique drug release pattern [217].

### 5.2. Hydrogel and Beads

Hydrogels are 3D networks of water-soluble polymers. They have received considerable attention as drug delivery systems, mainly because their highly porous structure permits loading of drugs into the gel matrix and subsequent drug release at a rate dependent on the diffusion coefficient of the molecule through the gel network. Furthermore, hydrogels swell without dissolving when in contact with water or other biological fluids and can easily be tuned by controlling the density of cross-links in the gel matrix [248].

Several works on carrageenan-based hydrogels for drug delivery applications have been published. Very different uses that include the production of ophthalmic [176] and floating drug delivery systems [177], antimicrobial wound dressings [178] and combined carrier systems such as lipid-hydrogel films [185], have been reported. In fact, given the physicochemical properties of this carbohydrate, which were already discussed, carrageenan is frequently combined with other compounds to develop hydrogel systems. For instance, the combination of carrageenan with gelatin [179,180], poloxamer 407 [181] and sodium carboxymethyl cellulose [182] have been reported regarding the production of hydrogel composites for drug delivery applications. As well, the use of carrageenan-based hydrogels in tissue engineering has been evaluated. A study reported that carrageenan hydrogel provided an adequate support for culture and differentiation of encapsulated human-adipose-derived stem cells in the regeneration of cartilage [183]. Another report showed that the injection of human nerve growth factor β delivered by collagen/nano-hydroxyapatite/ κ-carrageenan gels to sites of new bone formation can appreciably improve bone consolidation [184].

Carrageenan beads were also described as potential drug carriers systems [151,152]. Carrageenan/chitosan beads were reported to efficiently deliver sodium diclofenac in gastrointestinal fluid [153]. Interestingly, a formulation of solid-lipid beads based on carrageenan was shown to have the potential to mask the bitter taste of enrofloxacin and extend its release rate [154]. *Kappa*-carrageenan/polyacrylamide beads have shown a pH-responsive behavior and were proposed for targeting ketoprofen to the intestine. The particles assembled by electrostatic interaction and ketoprofen release was significantly increased when pH of the medium was changed from acidic (maximum of 10% release) to alkaline (about 90% release) [155]. Besides, the use of κ-carrageenan beads might also be effective in the field of tissue engineering, being reported to incorporate platelet derived growth factor [157].

While fucoidan beads have not been reported, the application of this polysaccharide in the production of hydrogels used for drug delivery purposes was reported to address the therapy of ischemic disease. Nakamura and co-workers [218] developed an injectable chitosan/fucoidan micro complex-hydrogel which was found effective at releasing fibroblast growth factor-2 *in vitro* and *in vivo*. After subcutaneous injection in mice, significant neovascularization and fibrous tissue formation were induced near the site of injection at 1 week, and the hydrogel was biodegraded and disappeared after 4 weeks. Another work reported the functionalization of 3D scaffolds with fucoidan. The scaffolds were loaded with vascular endothelial growth factor, which is intended to provide angiogenic activity in ischemic tissues. Functionalized scaffolds induced higher neovessel area and density, comparing with scaffolds without fucoidan [249]. Other studies have investigated the pharmaceutical/biomedical application of fucoidan-based hydrogels, mainly regarding the treatment of dermal burns [99,250] or generally addressing wound healing [251]. However, in those cases no drug molecules were encapsulated, the proper hydrogels being used for the intrinsic properties of fucoidan.

Ulvan has been demonstrated to enable the production of hydrogels, although the majority of works proposes applications such as ion exchange [252] and tissue engineering [133,219], which do not specifically address the association and release of drugs. One sole study reports an ulvan hydrogel aimed at drug delivery. Ulvan was cross-linked with 1,4-butanediol diglycidyl ether to form a 2D structure, which was loaded with dexamethasone as model drug. It was observed an initial steady release of the drug (~49% in 8 h) followed by slower and sustained release up to 14 days [223]. The same group has reported the preparation of ulvan beads for a posterior incorporation into polylactic acid hydrogels in a strategy of bone engineering. The beads were produced by complexation with chitosan and also associated dexamethasone. The production of the hydrogel and the incorporation of the beads involved supercritical fluids technology and, although drug release studies and cytotoxicity evaluation were performed, these comprised the whole system and not only the ulvan beads [246].

### 5.3. Other Drug Delivery Systems

Apart from the described delivery systems, which are considered those of more advanced technology in the field, other classes of systems also report the use of the polysaccharides being focused in this review. Conventional systems like tablets have been widely explored but an application as fibers, films and wafers also deserves reference.

Matrix tablets are widely accepted for sustained release, as they are simple and easy to formulate, although they are one of the most conventional formulations and usually are not the focus of the latest drug delivery developments. Ulvan has no reports as part of the composition of tablets, but fucoidan was included in one work. Nevertheless, its inclusion envisaged benefiting from the anti-inflammatory, anti-coagulant and anti-tumor activities of the polymer and not an application as matrix material [253]. Contrarily, controlled-release tablet matrices containing carrageenan have long been studied. A very complete assessment in this regard was recently performed [9] and the readers are directed to that review for further details. Since then, some other works have come to light, some of them with interesting approaches. As general information, there are several aspects influencing the release of drugs from carrageenan matrix tablets. These include the type of carrageenan, the used drugs, which establish different interaction with the polymer, the presence of other excipients in the matrix, pH and ionic strength of the dissolution medium, type of diluent used, the compression force and tablet dimensions [254,255,256]. The release mechanism from tablets with different composition (including carrageenan) and associating different drugs (theophylline and metoprolol succinate) was investigated in a recent work [257]. Yet another work was devoted to studying the behavior of several polysaccharides, including λ-carrageenan, in the release of atenolol from drug tablets [258]. The role played by drug/polymer interaction on the water uptake, swelling, drug dissolution and drug release was investigated in a study using λ-carrageenan. It was observed that different drugs give complexes with quite different characteristics of solubility and drug release kinetics. In another approach, carrageenan was associated with two drug models, diltiazem HCl and metoprolol tartrate, and the two studied complexes released the drug with different mechanism indicating two different drug/polymer interaction strengths (stronger interaction with diltiazem) [259]. Other recent developments included the use of carrageenan, combined with other matrix materials, to associate and release vitamin B2 [260] and lisinopril [261].

The use of carrageenan was also tested to produce oral granules, but those composed of gelatin and a derivative of this protein exhibited better properties regarding elasticity and swelling ability [262].

Another application deserving reference, which is also a nano-sized carrier, consists of fibers. To date, only carrageenan and ulvan have such a reported application in the context of drug delivery. *Iota*-carrageenan fibers demonstrated to associate a wide range of low solubility drug molecules, including benzocaine, furosemide, griseofulvin, hydrocortisone, ibuprofen, indomethacin, phenylephrine HCl, sulfapyridine and thymol. The selected compounds cover a range of structural variety and therapeutic indications. The carrageenan matrix was found to protect drugs from thermal degradation. Griseofulvin was the drug selected to perform release assays, evidencing a burst release (80% in 20 min) and a plateau reached up to 2 h [263]. Although no direct application in drug delivery was reported, this was suggested by authors preparing ulvan nanofibers from a poly(vinyl alcohol) (PVA)-ulvan blend, using an electrospinning technique. The fibers were described as having a high degree of orientation, which was attributed to the ulvan component [221].

In the same line, another work also proposed electrospinning as a technique to produce nanofibers from a blend of ulvan and polycaprolactone or polyethylene oxide. Again, the association of a drug was not reported, but only suggested [222]. In general, nanofibers find application at the level of tissue engineering and thus the encapsulation and release of drugs, growth factors or other molecules might be of interest.

Other systems deserving a reference are films and wafers, which have been produced with carrageenan and applied in buccal drug delivery. To our knowledge, neither fucoidan nor ulvan have similar applications. Films prepared by combining κ-carrageenan, poloxamer^®^ 407 and polyethylene glycol were loaded with ibuprofen (up to 0.8%, *w*/*w*) as model hydrophobic drug. Drug dissolution at a pH simulating that of saliva showed that amorphous ibuprofen released from the films (65% in 120 min) at a faster rate than the pure crystalline drug [188]. Another work reported that mucosal films based on κ-carrageenan, carboxymethyl cellulose and glycerol (1:2:3, *w*/*w*) provided sustained release of paracetamol (85%) and amoxicillin (71%) for 8 h in pH 6.5 buffer [190]. Similar works have demonstrated the ability of carrageenan-based buccal films to incorporate and release drugs [189,193]. Wafers prepared with κ-carrageenan and pluronic^®^ were also proposed for buccal delivery of paracetamol or ibuprofen. Drugs remained stable over 6 months and the release in a medium resembling salivary pH was gradual within 2 h (ibuprofen reached 75% after 120 min whereas paracetamol released 50% in the same time [198].

Similar systems have also been proposed for wound healing. Wound dressings are traditionally used to protect the wound from contamination, but they can be exploited as platforms to deliver bioactive molecules to wound sites. Unlike traditional dressings such as gauze and cotton wool that are passive agents in the wound healing process, advanced therapeutic dressings are designed to have biological activity either on its own or by releasing bioactive components incorporated within the dressing. The incorporated drugs can play an active role in the wound healing process, such as antimicrobial or anti-inflammatory agents, or by removing necrotic tissue and promoting tissue regeneration. In chronic wound management, where patients usually undergo long treatments and frequent dressing changes, a system that delivers drugs to a wound site in a controlled manner can improve patient compliance and therapeutic outcomes [264]. Advanced therapeutic dressings for effective wound healing, including films and wafers based on biopolymers, are described in detail in a review [265].

On this matter, a group reported polyethylene oxide (Polyox^®^) and κ-carrageenan based films as dressings for drug delivery to wounds [191,192]. The films were loaded with streptomycin and diclofenac for enhanced healing effects in chronic wounds. Drug loaded films showed a high capacity to absorb simulated wound fluid and also significant mucoadhesion, which is expected to allow effective adherence to and protection of the wound. As well, the films showed controlled release of both streptomycin and diclofenac for 72 h. Besides, these drug loaded films produced higher zones of inhibition against *Staphylococcus aureus*, *Pseudomonas aeruginosa* and *Escherichia coli* compared to the individual drugs zones of inhibition. The same research group also developed wafers of similar composition and loading the same drugs and a sustained release of both drugs was also observed in 72 h [199].

Although a few studies have reported the development of films [266,267,268,269] containing fucoidan as wound dressings, these systems were not addressed to deliver drugs. Actually, fucoidan was used to actively participate in the process of wound healing for its intrinsic properties.

## 6. The Application of Sulfated Polysaccharides in Targeted Drug Delivery

The targeted or site-specific delivery of drugs, as the name suggests, is a method of delivering drugs to a patient in a very specific manner that allows concentrating the drugs in the site of interest, while reducing its concentration in the remaining tissues [270]. This not only improves the inherent efficacy of drugs, but also reduces side effects. Targeted drug delivery is, therefore, a very attractive attainment, because it provides one of the most potential ways to improve the therapeutic effect of drugs.

Since it is very difficult for a drug molecule to reach its destination in the complex cellular network of an organism, the assistance of a drug carrier is usually required for this end. Drug delivery systems offer an intelligent approach for carrying and, at the same time, modulating the release and the absorption of the drug. In summary, they may tailor the response. However, their success is frequently limited by short residence times at the site of absorption or action. For this reason, it would be advantageous to have means to provide an intimate contact between the drug delivery system and these sites. In this context, microstructural design and chemical composition can be used to adapt the structure-activity relationship and tailor improved polymeric matrices. Various polymer architectures (linear, branched backbones) and combinations of polymers physically mixed (polymer blends or interpenetrating networks) or chemically bonded (copolymers) offer tremendous scope as carrier systems [271]. Moreover, because of the versatility of preparation methods and chemical structure of polymers, surface functionalities may sometimes be incorporated in the carriers. This facilitates additional attractive properties, such as the attachment of ligands that prolong the circulation of the drug carrier system in the blood stream, or the targeting of ligands for interaction with specific cell receptors [272]. Therefore, when designing a drug delivery system, it is important to consider, not only the polymer characteristics, but also the specific properties of target cells [273].

Various drug delivery systems at the nano- and micro-scale have been designed with specific features, enabling the targeting of different cell types. For an active targeting approach, carriers must either have a matrix composed of materials that act as targeting moieties themselves or incorporate surface ligands with selective affinity for specific receptors. Carriers comprising polysaccharides or simpler carbohydrates in their structure have been referred for this end [274], for instance to target cancer cells [275,276], cells at epithelial surfaces [277,278] or macrophages [279,280,281]. Sulfated polysaccharides have been shown to be endowed with specific features that enable this targeting ability. The targeting of macrophages has been one of the most referred activities for this class of materials. It is well-known that macrophages express a variety of cell surface receptors, including those specific for mannose, fucose, galactose and *N*-acetylglucosamine residues [282]. Therefore, carbohydrates may be used as specific recognition signals to target macrophages and trigger immune responses. As a matter of fact, several studies have investigated the targeting of the macrophage mannose receptor, using carbohydrate-based agents [283,284,285,286]. Besides surface ligands/moieties, macrophage targeting might be favored by adapting the size, surface charges and hydrophobicity of the carriers [287,288,289,290]. A recent review from our group has addressed these aspects [282].

Particulate systems are engulfed by macrophages usually via two main endocytic pathways, phagocytosis or pinocytosis [291]. There is a long list of macrophage surface receptors which are frequently categorized in three main groups: Toll-like receptors (TLR), non-TLR and opsonic receptors. The latter include complement receptors (integrins) and Fc receptors (immunoglobulin superfamily). The mentioned opsonic receptors are those working towards the phagocytosis and endocytosis of complement- or antibody-opsonized particles, respectively. Other complementary receptors, such as C-type lectin receptors, also play a role in endocytosis and phagocytosis [282], recognizing conserved carbohydrate structures, including mannose and galactose. Moreover, the mannose receptor on macrophage surface is reported as being capable of recognizing mannose, fucose, *N*-acetylglucosamine units and sulfated sugars [282,289,292]. Nevertheless, the scavenger receptors are those playing the major role in macrophage recognition of carrageenan and fucoidan, particularly scavenger receptor class A [282]. Interestingly, a study evaluated the effects of the functional groups located on microsphere surfaces upon the uptake by alveolar macrophages. Both polystyrene microspheres and cellulose microspheres were modified to exhibit sulfate, hydroxyl or carboxyl residues on the surface. All microsphere surfaces were found to be negatively charged and were effectively taken up by macrophages. Nevertheless, the functionalization of cellulose microspheres with the same negatively charged groups enhanced particle internalization by peritoneal macrophage cells compared to those microspheres with non-ionic hydrophilic surface [293].

Considering all the above, sulfated polysaccharides extracted from marine algae may be of potential use in stimulating the immune system or controlling macrophage activity. In fact, the importance of sulfate groups on the macrophage-stimulating activities of ascophyllan extracted from the brown algae *Ascophyllum nosodum* has been recently reported [294]. Regarding polymers, carrageenan and fucoidan appear to exert immunomodulatory activities in mammals, as they have been reported to modify the activity of macrophages [295,296]. Actually, carrageenan has been investigated to activate macrophages. This carbohydrate is known to have inflammatory activity and, thus, induce the recruitment of monocytes and macrophages [297]. Moreover, it was found that carrageenans may increase binding and killing activities of macrophages [298], which might be very useful in therapies requiring the elimination of macrophage intracellular pathogens, such as in tuberculosis and leishmaniasis. Likewise, the immunomodulatory effect of fucoidan was also reported. In this regard, a study performed on macrophages infected with strains of *Leishmania donovani* demonstrated that fucoidan significantly enhanced the production of pro-inflammatory cytokines IL-12 and TNF-α, while markedly attenuated the level of IL-10 and TGF-β (anti-inflammatory cytokines) [299]. Another study revealed that fucoidan functionalized with acetyl residue induces macrophage activation through membrane receptors (toll-like receptor 4, CD14, scavenger and mitogen-activated protein kinase receptors) signaling pathways. The unique structural features of acetyl-fucoidan were suggested to play a relevant role in the activation process [300]. Nevertheless, several reports have suggested that fucoidan appears to modulate macrophage functions by inhibition rather than activation, acting as an anti-inflammatory agent [104,301,302]. The literature thus apparently provides contradictory information, but it is important to recall that, apart from the complexity of macrophage systems and actions, different fucoidan molecules might be used that result in rather different (and inclusive opposite) outcomes. Other research groups have reported the immunomodulating activities exhibited by fucoidan depending on variations of their structural features [104,105].

The structural characteristics of ulvan also suggest a potential immunomodulating property for this polysaccharide, which has been observed both in nature [303] and towards a macrophage cell line [304]. As mentioned in Section 4.2, ulvan is mainly formed by glucuronic acid and sulfated rhamnose, a disaccharide that resembles glycosaminoglycans, such as hyaluronan and chondroitin sulfate, regarding the content in glucuronic acid and, in the latter, also sulfate groups. All this makes ulvan worthy of investigation. Indeed, ulvan polysaccharides extracted from the seaweed *Ulva rigida* induced the expression of IL-1β, an inflammatory marker, in turbot peritoneal leucocytes. The overall results indicated that sulfate groups were required to induce this activation response [305]. These results were confirmed in another study, where the activity of murine macrophages (RAW264.7), evaluated through the production of inflammatory cytokines and receptors, and nitric oxide, was demonstrated to be modulated by the same ulvan polysaccharides. Furthermore, the presence of sulfate groups in the molecules was suggested to be determinant to obtain the activation effects, as the desulfation of the molecules decreased considerably the stimulatory capacity of the acidic polysaccharides [116].

In most of the current approaches of drug delivery, drug molecules are incorporated in carriers. Taking into account the information exposed above, the application of sulfated polysaccharides in carrier structures could be beneficial regarding the interaction with cells. However, only few reports aimed at a cell targeting strategy propose carriers based on sulfated polysaccharides in general, and the number becomes even narrower when the three considered polysaccharides are focused. In this regard, although not addressing the delivery of a drug molecule, a study on the interaction of fucoidan-coated isobutylcyanoacrylate nanoparticles with both macrophages (J774) and fibroblasts (NIH-3T3), indicated that the interaction is modulated by the presence of fucoidan in macrophages, but not in fibroblasts [244].

The potential for cell targeting strategies is identified, mainly owing to the unique structural features of the addressed sulfated polysaccharides. Nevertheless, to our knowledge, this potential has never materialized into the design of carriers based on carrageenan, fucoidan or ulvan aimed at providing cell targeting in a drug delivery strategy. A higher investment of researchers in this regard is expected in the coming years, as the physicochemical properties of these carbohydrates enable the ability for interaction with other compounds, either drugs, proteins or other polymers. Moreover, the presence of sulfate groups along with the proper carbohydrate-based structure may be used as particular recognition signal to specifically deliver biological active components.

## 7. Conclusions

Usually, the chemical structure of polymers is the driving force for the many applications described. With sulfated polysaccharides and, namely carrageenan, fucoidan and ulvan, the same observation applies, as their biological and physicochemical properties, naturally resulting from the specific chemical structure, have dictated the registered advancements in drug delivery strategies. While in the past excipients were traditionally included in the formulations as inert substances whose role only consisted of aiding production processes, in the last decades the paradigm has changed and they are now approached as multifunctional excipients. This means that several functions apply and, in the case of the specific polysaccharides under review, these entail from the stabilization and control of the release, to providing biocompatible properties and targeting moieties, just to mention some. Applications in the development of particulate carriers, either at the micro- or nanoscale, have been gathering the greatest interest. However, the production of hydrogels and beads, and also matrix tablets fibers and films, has also been described and explored in this review. Carrageenan is the most described polymer in all the applications, while ulvan is by far that with lower number of reported uses. This is certainly a result of the difficult characterization of the polymer, because of its irregular composition, along with its apparent complexity. Applications of these polysaccharides at the level of cell targeting will certainly be further explored in the future, as therapeutic approaches to local and systemic diseases might be envisaged. However, in order to take the maximum benefit from the potential of these materials, there is a strong need to dedicate research to a deeper knowledge on the chemical structure, permitting the rigorous definition of the basic structures, as well as the inherent physicochemical properties. In parallel, the optimization of extraction procedures, providing more pure biomaterials, is also a desired achievement. In fact, the uncertainty about structures and the difficulties in extraction are definitely the strongest limitations regarding the proposal of applications, thus preventing the progression of these materials to more advanced therapeutic solutions. Additionally, long-term toxicity assays will be needed to effectively evaluate the possibility of using the materials in drug delivery approaches. As a whole, the potential of the materials in several applications has been indicated. Basic characteristics remain, however, to be clarified and characterized, so that the applications can be materialized into more intense progression.

## Figures and Tables

**Table 1:** Description of several characteristics of carrageenan, fucoidan and ulvan.

Sulfated Polysaccharide	Marine Algae Group	Main Genera	Molecular Weight (kDa)	Solubility in Water	Viscosity (cps, in Water)	pH in Aqueous Solution	References
Carrageenan	Rhodophyceae	*Chondrus* *Euchema* *Furcellaria* *Gigartina* *Hypnea* *Iridae* *Kappaphycus*	100–1000	* κ-, ι- and λ-carrageenan soluble at 80 °C	5–800 (1.5% *w*/*v*, 75 °C)	7.0–10.0	[2,7,8,9,10]
Fucoidan	Phaeophyceae	*Analipus* *Chorda* *Dictyota* *Fucus* *Kjellmaniella* *Pelvetia* *Sargassum* *Undaria*	10–950	10 mg/mL (*F. vesiculosus*)	n.a.	n.a.	[11,12,13,14,15,16,17,18,19]
Ulvan	Chlorophyceae	*Enteromorpha* *Ulva*	1.14 to > 2 × 10^6^	n.a.	18–100 (1.6% *w*/*v*, *Ulva* spp.)	7.5 (*Ulva* spp.)	[20,21,22]

* Further solubility conditions in Reference [10]; n.a.: not available.
